# Transcriptome and methylome of the supraoptic nucleus provides insights into the age-dependent loss of neuronal plasticity

**DOI:** 10.3389/fnagi.2023.1223273

**Published:** 2023-08-30

**Authors:** Derick Thompson, Abiodun E. Odufuwa, Catherine A. Brissette, John A. Watt

**Affiliations:** Department of Biomedical Sciences, School of Medicine and Health Sciences, University of North Dakota, Grand Forks, ND, United States

**Keywords:** supraoptic nucleus, magnocellular neurons, neural regeneration, neural plasticity, transcriptome, DNA methylation, axonogenesis, MHCI

## Abstract

The age-dependent loss of neuronal plasticity is a well-known phenomenon that is poorly understood. The loss of this capacity for axonal regeneration is emphasized following traumatic brain injury, which is a major cause of disability and death among adults in the US. We have previously shown the intrinsic capacity of magnocellular neurons within the supraoptic nucleus to undergo axonal regeneration following unilateral axotomization in an age-dependent manner. The aim of this research was to determine the age-dependent molecular mechanisms that may underlie this phenomenon. As such, we characterized the transcriptome and DNA methylome of the supraoptic nucleus in uninjured 35-day old rats and 125-day old rats. Our data indicates the downregulation of a large number of axonogenesis related transcripts in 125-day old rats compared to 35-day old rats. Specifically, several semaphorin and ephrin genes were downregulated, as well as growth factors including FGF’s, insulin-like growth factors (IGFs), and brain-derived neurotrophic factor (BDNF). Differential methylation analysis indicates enrichment of biological processes involved in axonogenesis and axon guidance. Conversely, we observed a robust and specific upregulation of MHCI related transcripts. This may involve the activator protein 1 (AP-1) transcription factor complex as motif analysis of differentially methylated regions indicate enrichment of AP-1 binding sites in hypomethylated regions. Together, our data suggests a loss of pro-regenerative capabilities with age which would prevent axonal growth and appropriate innervation following injury.

## Introduction

Traumatic brain injuries (TBIs) are one of the most common causes of disability and death of adults in the US. In 2014, there were 2.8 million TBI events that lead to emergency department visits, hospitalizations, or deaths (Centers for Disease Control and Prevention [CDC], 2019). In 2020, there were 64,000 TBI-related deaths in the US, representing 176 deaths per day (Centers for Disease Control and Prevention [CDC], 2022). The most severe of these events that lead to hospitalization or death correlate significantly with increasing age (Taylor et al., 2017; Centers for Disease Control and Prevention [CDC], 2022). Furthermore, the rate of a full recovery in adults following a moderate or severe TBI are poor – with a less than 50% chance of a favorable recovery in individuals 30 years or older (Dhandapani et al., 2012). The increasing inability to recover from a brain injury with age is not a new phenomenon and is shared across many mammalian species. These patterns represent a major need to fully understand the regenerative capacity of the mature mammalian central nervous system (CNS) following injury.

In our previous studies, the magnocellular neurosecretory system was shown to exhibit this maturational loss of regenerative capacity (Watt and Paden, 1991; Watt et al., 1999; Askvig and Watt, 2019). This system is comprised of two regions of interest – the supraoptic nucleus (SON) and the posterior pituitary gland, or neural lobe (NL). The SON is found bilaterally and adjacent to the optic chiasm, and contains magnocellular neurons (MCNs) that project long axons to the NL that each branch into thousands of nerve terminals (Silverman and Zimmerman, 1983). Through this, MCNs produce oxytocin and vasopressin that travel and secrete into the NL where it is then released into the bloodstream (Silverman and Zimmerman, 1983). Astroglia are another major cell type within the SON. Under homeostatic conditions, astroglial processes can be observed separating MCN somata and their ventral dendrites from one another (Hatton, 2004). This astrocytic coverage allows for the modulation of neurotransmitter levels within the extracellular space, specifically glutamate, and enabling the regulation of neuronal activity (Oliet, 2002; Oliet and Bonfardin, 2010). The SON displays pronounced affinity to structural and morphological plasticity under important physiological events and conditions: during parturition and lactation, dehydration and salt-loading, and in our prior research, during traumatic unilateral lesion of MCN axons. These stimuli result in structural changes in neuron-glial interactions, namely the reduction of astrocytic processes in contact with neurons, and enhanced communication and activity of neurons (Tweedle and Hatton, 1977, 1984; Hatton and Tweedle, 1982; Cobbett and Hatton, 1984; Theodosis and Poulain, 1984; Watt and Paden, 1991; Hatton, 2004; Johnson et al., 2015).

Our previous research showed that unilateral lesion of SON-MCN axons leads to a collateral sprouting of neurosecretory axons within the NL stemming from the contralateral, uninjured SON in 35-day old rats (Watt and Paden, 1991; Watt et al., 1999). However, this phenomenon is lost with age, as 125-day old rats exhibit no regenerative sprouting as evidenced by a lack of restoration to basal levels of nerve terminals in the NL (Askvig and Watt, 2019). Traumatic brain injuries encompass a large range of injuries that can be generally placed into two categories: penetrating and non-penetrating. With the intrinsic capacity of the supraoptic nuclei plasticity, we can better understand the age-dependent recovery following a penetrating injury through the use of the aforementioned lesion model. Where many studies of CNS injury focus on the physiological changes at the site of the injury, the use of the SON unilateral injury model allows us to investigate both the site of injury and the contralateral non-injured site that undergo compensatory effects. Following unilateral axotomization of MCNs in the SON, the number of ipsilateral MCNs are drastically reduced, whereas the contralateral MCNs become hypertrophic and undergo axonal sprouting (Watt and Paden, 1991). Furthermore, in studies using organotypic cultures of the SON, in which both nuclei are lesioned, it has been observed that neuronal survival and process outgrowth can be induced following CNTF administration via the MAPK and PI3K pathways (Askvig and Watt, 2015). Investigating the age-dependence of this phenomenon as well as the cellular and molecular differences between the ipsilateral and contralateral SONs during lesion would provide great insight into the mechanisms of recovery following injury.

In this study, we investigated the molecular alterations that occur with age in uninjured SONs. We hypothesize that with age, the magnocellular neurosecretory system changes to, and maintains, a molecular profile that is conducive to the inhibition of neuronal plasticity. More specifically, we hypothesize that a distinct lack of axonal growth factors facilitates this inability for axonal sprouting. As such, our goal was to investigate changes in the transcriptome and DNA methylome between 35-day and 125-day old uninjured rats in order to determine the establishment of unique age-related molecular profiles in the SON that may underlie the loss of regenerative capacity following injury. Thus, we performed RNA-seq and demonstrated a robust downregulation of genes related to axonogenesis and axon guidance that include several families of growth factors, semaphorins, and ephrins. Even though we considered our 125-day old adult rats to be “mature,” and not “aged,” we anticipated a minor increase in inflammatory profiles similarly seen in more advanced ages. However, we found a very specific increase in the expression of genes involved in antigen processing and presentation, specifically that of MHCI, and a lack of inflammatory cytokines. Next, we determined mechanisms of transcriptional regulation via DNA methylation. Our analysis indicates an enrichment in differential methylation that overlapped with genes involved in axonogenesis. Furthermore, motif enrichment analysis suggests that the regulation of MHCI pathway genes occurs through the hypomethylation of motifs for the transcription factor activator protein 1 (AP-1). Here we present our findings that suggest that age-dependent changes in DNA methylation influences the transcriptional profile of the SON toward inhibition of axonal sprouting and plasticity.

## Materials and methods

### Animals

Male Sprague-Dawley rats were purchased from Envigo (Indianapolis, IN, USA), and housed at the University of North Dakota Center for Biomedical Research Facility. All animal experiments complied with ARRIVE guidelines and were carried out in accordance with the National Research Council Guide for the Care and Use of Laboratory Animals and protocols approved by the University of North Dakota (UND) Institutional Animal Care and Use Committee (protocol number: 1905-7). Animals used in this study were 34–36 days of age (for “35-day old” rats), and 124–126 days of age (for “125-day old” rats).

### Tissue collection

Rats were anesthetized briefly using isoflurane (Covetrus, NDC: 11695-6777-2) and perfused transcardially with PBS. In order to maintain RNA and DNA integrity for sequencing and PCR, the tissue was unfixed. Following perfusion, the brain was removed and the region of the brain containing the SON was isolated by taking an approximately 3 mm thick coronal section. This and subsequent sections were maintained in ice chilled HBSS (ThermoFisher, Gibco; Cat. #14185052) and supplemented with sodium bicarbonate. This coronal section was further sectioned into approximately 200 μm serial sections using a vibratome (Ted Pella, Inc.; Cat. #11000-00137). Sections that contain the SON are approximately between bregma −0.8 and −1.8 mm. Sections containing the SON were further processed – using a light dissecting microscope, the SON can be located bilaterally and adjacent to the optic chiasm. A 1 mm tissue puncher was used to specifically dissect out the SON. The SON was then snap-frozen in liquid nitrogen prior to storage at −80°C.

### RNA and DNA isolation

Tissue from four separate animals were pooled together prior to RNA and DNA isolation, and represent a single biological replicate. This was required in order to obtain sufficient biological material of high quality. For RNA-sequencing, four biological replicates were used for each group. For nanopore sequencing, three biological replicates were used for each group.

For RNA, samples were ground under liquid nitrogen, after which 1 ml of TRIzol reagent was added (Invitrogen; Cat. #15596026). RNA was isolated via phenol-chloroform extraction and column purification by RNeasy Mini kit (Qiagen; Cat. #74106) according to the manufacturer’s instructions. In brief, 200 μl of chloroform was added to the TRIzol suspensions and centrifuged to obtain phase separation. The aqueous phase was removed and 70% ethanol was added to it. This was then placed in a Qiagen RNeasy Mini column and further washed and processed according to Qiagen’s instructions. Genomic DNA was degraded with RNase-free DNase Set (Qiagen; Cat. #79254).

DNA was isolated by ethanol precipitation. In brief, samples were ground under liquid nitrogen where 180 μl of Buffer ATL (Qiagen; Cat. #1014758) and 20 μl of Proteinase K (Qiagen; Cat. #1017738) were added to the sample for lysis and incubated for 1 h at 56°C. A total of 4 μl of RNase A (100 mg/ml; Qiagen; Cat. #1007885) was added for the digestion of RNA and Buffer ATL to bring the total volume to 500 μl. A total of 500 μl of phenol:chloroform:isoamyl alcohol (25:24:1; Sigma; Cat. #77617) and centrifuged. The resulting aqueous phase was removed (approximately 500 μl) and placed in a new tube where 5 μl of glycogen (20 mg/ml; Invitrogen; Cat. #10814010), 50 μl 5M ammonium acetate, and 550 μl isopropanol were added. This was then incubated overnight at −20°C, after which it was thawed and centrifuged to form a pellet. This pellet was washed and centrifuged twice with 70% ethanol and air dried for 5 min. The final DNA pellet was resuspended in Buffer EB (Qiagen; Cat. #19086).

### Library construction and sequencing

RNA integrity was assessed using an Agilent 2100 Bioanalyzer with RNA 6000 Nano Kit (Agilent; Cat. #5067-1511). All samples had an RNA-integrity number between 8.8 and 10.0. NEBNext Poly(A) mRNA Magnetic Isolation Module (NEB; Cat. #E7490S) and NEBNext Ultra II RNA-seq library kit for Illumina (NEB; Cat. #E7770S) was used for mRNA enrichment and library construction, respectively. Library quality was assessed by bioanalyzer with Agilent High Sensitivity DNA kit (Agilent; Cat. #5067-4626) and library concentration was determined with a BioTek BFLx800 fluorescence microplate reader with the Quant-iT PicoGreen dsDNA Assay kit (ThermoFisher; Cat. #P11496). Samples were sequenced on an Illumina HiSeqX and 150 bp paired-end sequencing across 3 lanes was performed. The reads per sample ranged from 35.5 million to 61.0 million reads, with a mean depth of 45.3 million reads.

Genomic DNA was assessed using an Agilent 4200 Tapestation Genomic DNA screentape (Agilent; Cat. #5067-5365). All samples had a DNA-integrity number between 9.5 and 9.7. DNA was then fragmented using a Covaris g-TUBE (Covaris; Cat. #520079). Libraries were prepared using the Oxford Nanopore Ligation Sequencing Kit (Oxford; Cat. #SQK-LSK109). DNA methylation was assessed by nanopore sequencing with a GridION Mk1 Nanopore by Oxford Nanopore Technologies using 6 MinION flow cells.

### RT-qPCR

RT-qPCR was performed as a technical validation method for RNA-seq. Thus, samples used for RT-qPCR were the same used for RNA-seq. Selected transcripts were confirmed using Quantitect primer assays obtained from Qiagen – *actb* (Cat. #QT00193473), *epha5* (Cat. #QT00386666), *sema3a* (Cat. #QT00192941), *tapbp1* (Cat. #QT00189819), *rt1-a2* (Cat. #QT00443240), *rt1-m3-1* (Cat. #QT00194054), *bdnf* (Cat. #QT00375998), *gfap* (Cat. #QT00195517), *oxt* (Cat. #QT01809752), and *avp* (Cat. #QT00184849). Reverse transcription of RNA to synthesize cDNA was performed using Qiagen’s First Strand Kit (Qiagen, Cat. #330404). PCR reactions were performed in accordance to the manufacturer’s instructions with the use of Qiagen’s Quantitect SYBR Green PCR Kit (Qiagen; Cat. #204143). Relative gene expression between 125- and 35-day old samples were determined using the 2^–ΔΔCT^ method. Expression levels were normalized to *actb*. All samples were analyzed in triplicate. Statistical analysis was performed using an unpaired Student’s *t*-test. This was followed by a Benjamini–Hochberg correction (FDR) to account for multiple testing.

### RNA data analysis

Quality for raw and trimmed reads were assessed using FastQC v0.11.9. Trimming of adaptors was performed by trimmomatic v0.39. Reads were aligned to the rat (rn7) assembly using Hisat2 v2.2.1 and sorted and indexed by Samtools v1.13. Annotation to assign reads to gene features was performed using featureCounts (Subread v2.0.1). Differential gene expression analysis was performed using DESeq2 v1.34.0. Genes were considered differentially expressed in 125-day old rats compared to 35-day old rats at a false discovery rate (*p.*adjust) of ≤0.05, a basemean >20, and a fold-change > 1.5.

Functional analysis (gene ontology) of biological processes was performed using the package clusterprofiler v4.2.2. To gain better biological context, upregulated and downregulated genes were separated for this. Significance threshold for biological process enrichment was *p.*adjust ≤ 0.01. Kyoto Encyclopedia of Genes and Genomes (KEGG) pathway analysis was performed on all differentially expressed genes (DEGs) using Signaling Pathway Impact Analysis (SPIA) v2.46.0. SPIA allows for the determination of KEGG pathway enrichment, and by implementing fold-change and topology of the pathway, it can determine if a pathway is activated or inhibit. Statistical significance was determined at pGFDR ≤0.05. Pathview v1.34.0 was used to overlay enrichment results with visualizations of pathways from KEGG.

### DNA methylation data analysis

Fast5 and fastq reads were indexed using Nanopolish v0.14.0. Reads were aligned to the rat (rn7) assembly using minimap2 v2.24 and sorted and indexed with Samtools v1.6. Methylation calling of reads was performed again by Nanopolish and methylation frequency was determined by the “calculate_methylation_frequency.py” script from Nanopolish. Bsseq v1.30.0 was used to create and smooth BSseq object required for differential testing. Testing and calling of differentially methylated loci (DML) was performed using DSS. DMLs were called using the callDML function of DSS with default settings except for *p*.threshold = 0.001. Differentially methylated regions (DMRs) were prioritized over DMLs for downstream analysis – a DML consists of a single differentially methylated CpG, whereas a DMR requires and consists of multiple CpGs. Our analysis required a minimum of three CpGs to be considered a DMR, which is the default setting for callDMR. This is a more conservative approach which strengthens the correlation between our observed DNA methylation and transcriptional changes. The callDMR function was performed under default settings except for *p*.threshold = 0.01.

For functional and pathway analysis, distal intergenic DMRs were removed – the current method for mapping DMLs and DMRs outside of gene bodies (i.e., distal intergenic) is based solely on proximity; the closest gene to a DMR is thus mapped to that DMR resulting in DMRs being mapped to genes that are millions of bps away. We felt it pertinent to only focus on DMRs that can be directly associated with a gene. Functional and pathway analysis was performed by the same packages as RNA-seq results, clusterprofiler and SPIA, respectively. Many genes contained both hyper and hypomethylated DMRs and thus separating them resulted in similar overlapping profiles, therefore they were not separated in this analysis.

Motif analysis was performed using the findMotifsGenome.pl function of HOMER. All DMRs, including distal intergenic DMRs were used for this analysis. Previously we ignored distal intergenic DMRs as they lacked direct association to genes; however, in this analysis, we are only focused on the DMRs themselves and not their gene association. Furthermore, many regulatory elements, such as enhancers, exist outside of the gene body, so the inclusion of distal intergenic DMRs is important to understand this outcome. Statistical significance of motif enrichment is *p*-value ≤ 0.05.

## Results

The goal of this study was to investigate alterations in gene transcription and DNA methylation with age in healthy non-injured rats in order to determine a mechanistic understanding for the loss in regenerative capacity following TBI, as observed in our prior experiments. To do this, we dissected SON samples from healthy, non-injured 125-day old rats and compared them to equivalent 35-day old rats to analyze the transcriptome via RNA-seq and DNA methylation via Nanopore long-read sequencing.

### Transcriptome characterization of the SON

**FIGURE 1:**
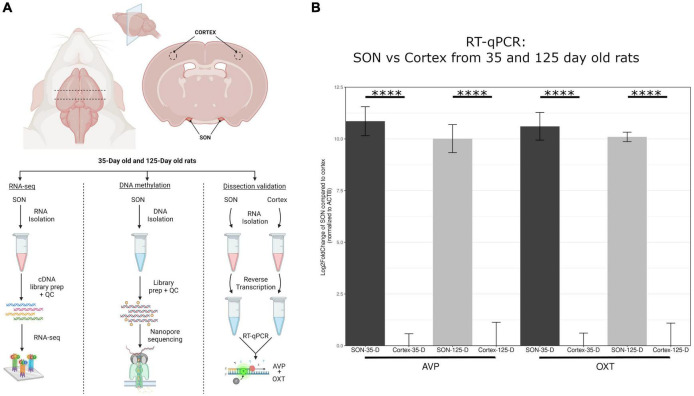
Overview of methodology. **(A)** This highlights the dissection methods used to isolate the SON. Four biological replicates for 35-day old and 125-day old rats were used for RNA-seq, and three biological replicates for each group for DNA methylation sequencing via nanopore. **(B)** RT-qPCR of *avp* and *oxt* was performed to determine accuracy of SON microdissection in comparison to cortex. Compared to cortex, *avp* and *oxt* was elevated approximately 1000-fold (log2FC = ∼10) for both age groups. Error bars represent SD. The symbol “^****^” denotes *p*-value < 0.0001.

**FIGURE 2:**
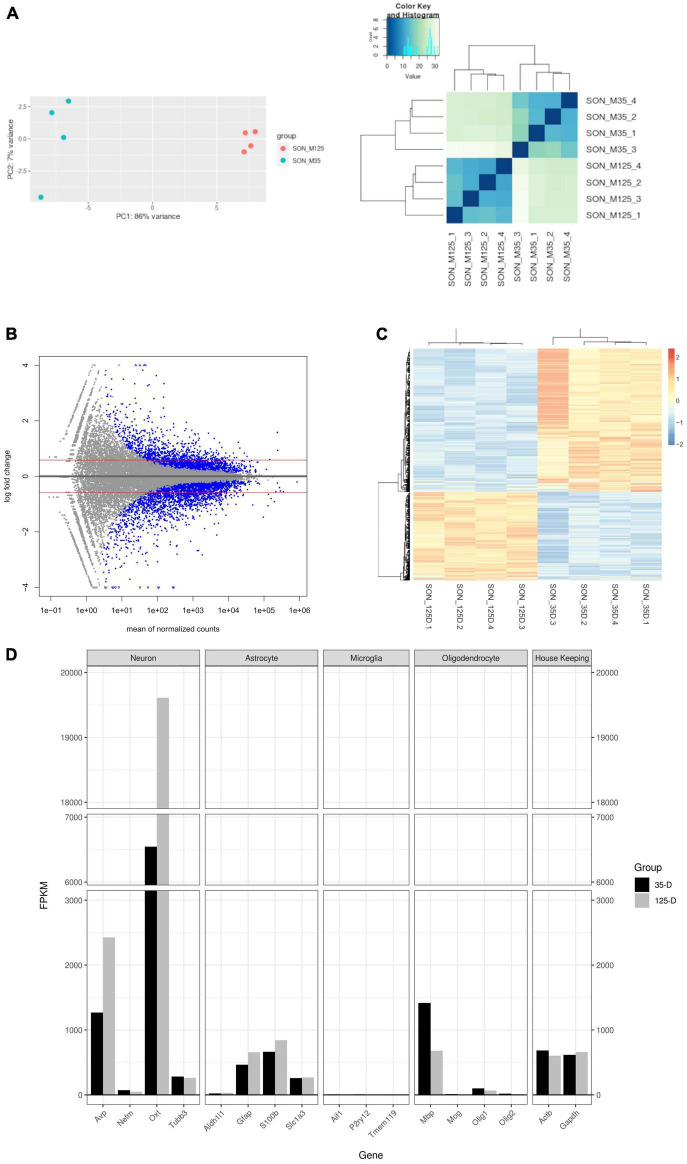
Characterization of RNA-seq data. **(A)** PCA plot and hierarchal clustering of biological replicates indicate clustering between age groups. **(B)** MA plot of RNA-seq data. Blue dots indicate statistically significant genes (*p*.adjust ≤ 0.05). Genes were considered differentially expressed with a *p*.adjust ≤ 0.05, basemean >20, and a fold-change ≥1.5 (red line). A total of 707 genes were upregulated and 1,145 downregulated. **(C)** A heatmap and Spearman correlation of DEGs, indicating that DEGs remain clustered by group. **(D)** A barplot of select gene markers (FPKM) indicating relative abundance of transcript levels.

The microdissection of the SON is performed based on anatomical apposition to the optic chiasm, as outlined in the methods section. As such, it was necessary to determine accuracy of these tissue microdissections (Figure 1A). The SON is one of two nuclei that produces oxytocin (OXT) and vasopressin (AVP), the other being the paraventricular nucleus (PVN), which is found adjacent to the third ventricle and distal enough to thus not be a potential source of contamination. To determine the accuracy of our SON microdissections, we analyzed the enrichment of *Oxt* and *Avp* from these tissue samples. To do this, we performed RT-qPCR from RNA isolated from these samples and compared them to cortex samples. For 35-day and 125-day SON samples, an approximate average of 10.5 Log2FC was observed for *Oxt* and *Avp*, representing a 1,000× enrichment between SON and cortex samples (Figure 1B). Our RNA-seq data corroborates this as seen by a large FPKM (Fragments Per Kilobase of transcript per Million mapped reads) values for *Oxt* and *Avp* relative to other genes (Figure 2D).

Transcriptional analysis was performed by RNA-seq. Principle component analysis and hierarchal clustering show distinct clustering of replicates within their respective age groups (Figure 2A). A total of 1,852 DEGs were found – 707 upregulated and 1,145 downregulated (Figure 2B and Supplementary File 1). Similarly, a heatmap and Spearman correlation of DEGs highlight clustering between age groups and gene expression (Figure 2C). Supplementary File 2 contains the statistical results of all genes between 35-day old and 125-day old rats and the normalized and FPKM reads for each replicate. RT-qPCR of selected genes was performed to validate RNA-seq results (Supplementary Figure 1).

Figure 2D shows quantification of cell-specific genes of the SON with FPKM values. As expected, the most abundantly expressed genes are from the MCNs which secrete oxytocin and vasopressin. Relatively, MCNs and astrocyte transcripts were the most abundant and showed increased expression with age. Conversely, microglia and oligodendrocyte transcripts were less prevalent and decreased in expression with age. Myelin basic protein (*Mbp*) was observed to have a much larger expression compared to other oligodendrocyte transcripts – this is most likely due to splice variants (Golli-*Mbp*) which are commonly expressed in neurons (Landry et al., 1996; Siu et al., 2015).

### Key genes for axonal plasticity are downregulated with age in the supraoptic nucleus

**FIGURE 3:**
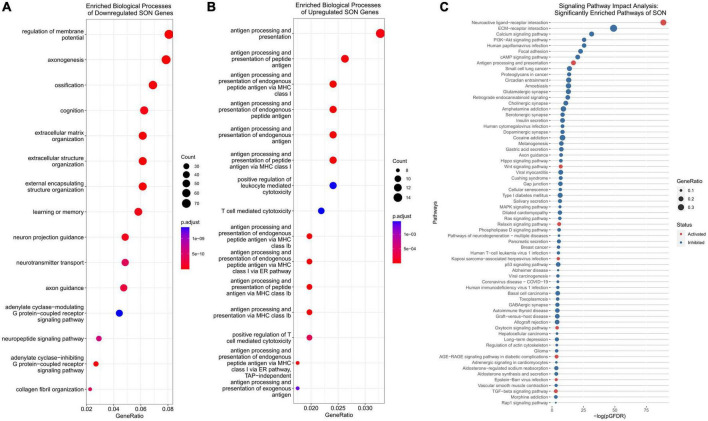
Functional analysis of DEGs. **(A)** Functional analysis of downregulated genes indicate an enrichment of axonogenesis, neuron projection guidance, and axon guidance biological processes. **(B)** Functional analysis of upregulated genes indicate an enrichment of antigen processing and presentation associated biological processes, including T-cell mediated cytotoxicity. **(C)** Signaling pathway impact analysis (SPIA) allows for the analysis of DEGs for KEGG pathway enrichment. SPIA utilizes log2FC and pathway topology to determine if a pathway is inhibited (blue) or activated (red). Antigen processing and presentation is activated, and axon guidance inhibited.

**FIGURE 4:**
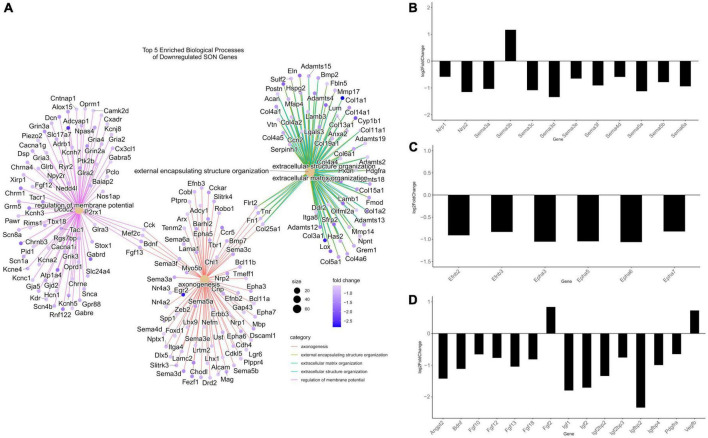
Differentially expressed genes related to axonogenesis. **(A)** A CNET plot of the top five enriched biological processes of downregulated genes. Genes related to axonogenesis, as well as genes related to extracellular matrix organization can be seen. **(B)** Barplot of semaphorin-related DEGs from RNA-seq, plotted by log2FC. The majority of semaphorins and neuropilins (*nrp1/2*) are downregulated. **(C)** Barplot of ephrin-related DEGs from RNA-seq, plotted by log2FC. Ephrins and their receptors were observed to be downregulated. **(D)** Barplot of growth factor DEGs from RNA-seq, plotted by log2FC. The majority of differentially expressed growth factors were downregulated. All genes within barplots were differentially expressed (*p*.adjust ≤ 0.05, basemean >20, and a fold-change ≥1.5).

In our previous studies, we have shown that in young (35-day old) rats, unilateral lesion of SON MCN axons leads to collateral axonal sprouting from the contralateral SON. However, this phenomenon is lost with age and is not observed in mature rats (125-day old). Following RNA-seq and differential gene expression analysis, we performed gene ontology (GO) over-representation analysis of biological processes to understand the molecular mechanisms that may underlie this shift in neuronal plasticity. Over-representation analysis of downregulated genes indicated 508 statistically significant (*p*.adjust < 0.01) biological processes (Supplementary File 3). Among the most enriched were several processes that relate to the modulation of neuronal plasticity, including axonogenesis (GO:0007409), axon guidance (GO:0007411), and neuron projection guidance (GO:0097485) (Figure 3A). A cnetplot of the top 5 enriched biological processes of downregulated genes highlight several key genes within axonogenesis that are shared across these processes (Figure 4A). Semaphorins (SEMAs), in conjunction with their receptors, the plexins and neuropilins protein families, play a major role in the growth of axons (Carulli et al., 2021). Specifically, their role is to aid in the guidance of axons by acting as repulsive cues for neurite growth and allow axons to find their appropriate target (Carulli et al., 2021). As such, we observed several genes within the semaphorin family and the neuropilin-1/2 receptors to be downregulated (Figure 4B). The plexin family of receptors for semaphorins showed constitutive expression and general downregulation but were not statistically significant. Similarly, several genes that encode for ligands and receptors of the ephrin family were found to be differentially downregulated with age (Figure 4C). Like SEMAs, ephrins play a key role in axon path finding, and are generally upregulated following injury (Yang et al., 2018). The WNT family of genes is a well-studied pathway that regulates many biological processes throughout early development and adulthood. More recent research has implicated WNT in traumatic brain injuries (Menet et al., 2020). We observed genes within the WNT signaling pathway associated with axonal guidance to be significantly downregulated. Signaling pathway analysis (SPIA) corroborates this loss in axonal guidance cues – the axon guidance pathway (KEGG rno04360) is observed to be significantly enriched and inhibited (Figure 3C and Supplementary File 3). SPIA utilizes enrichment analysis in combination with topological significance (e.g., location of a gene within the pathway) to determine global biological and statistical significance. As such, the analysis allows for the determination of activation or inhibition of specific pathways provided by KEGG (Figure 3C). The loss in appropriate guidance cues in aged rats would suggest the inability for MCNs in the SON to properly initiate, extend, and terminate in the posterior pituitary gland.

In addition to the loss of repulsive axon guidance cues, we observed a downregulation of several growth factor genes (Figure 4D). Fibroblast growth factors (FGFs) have been shown to be important for embryonic development and adult homeostasis, and many have been implicated in tissue repair following injury (Chen et al., 2022). Four FGF genes were significantly downregulated (*Fgf10*/*12*/*13*/*18*), while *Fgf*2 was found to be upregulated. Similarly, genes within the insulin-like growth factor (IGF) family were found to be downregulated, these include *Igf1/2* and several IGF-binding proteins (IGFBPs). These IGF proteins have been implicated as important players for neuronal survival and plasticity in brain insults (Mangiola et al., 2015; Allodi et al., 2016; Nieto-Estévez et al., 2016; Khan, 2019; Beletskiy et al., 2021). The brain-derived neurotrophic factor (*Bdnf*), which plays a crucial role in survival and plasticity of neurons, was also found to be downregulated (Miranda et al., 2019). Aside from having direct impacts on axonogenesis related pathways, these growth factors are capable of modulating the PI3K-Akt pathway. We observed, via SPIA, that the PI3K-Akt pathway (KEGG: rno04151) was inhibited, primarily through the downregulation of these growth factors, and the downregulation of extracellular matrix (ECM) genes and receptors (Supplementary Figure 2). In fact, our analysis indicates that ECM organization (GO:0030198) was enriched within downregulated DEGs, and that ECM-receptor interaction (KEGG: rno04512) and focal adhesion (KEGG: rno04510) pathways were inhibited (Figures 3A, C). Within these pathways, many genes within the laminin, collagen, and ADAM/ADAMTS family were found to be downregulated, suggesting that alterations to the ECM and focal adhesion molecules may play a significant role in the loss of axonal regeneration within our model system (Figure 4A).

### MHC class I and peripheral immune cytotoxicity were robustly upregulated with age

**FIGURE 5:**
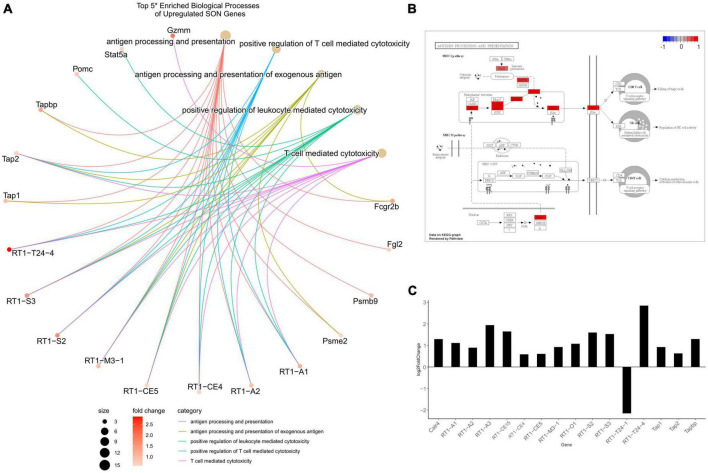
Differentially expressed genes related to MHCI. **(A)** A CNET plot of top 5 enriched biological processes of upregulated genes – (*) as antigen presentation and processing made up the top 11, the top 5 after these were selected to highlight a greater range of processes. RT1-related genes are the major component for MHCI, with associated processing genes including *tap1/2* and *tapbp* being upregulated. **(B)** View of the antigen processing and presentation KEGG pathway. This indicates that only MHCI related genes were upregulated, while MHCII remained unchanged. **(C)** A barplot of MHCI-related genes from RNA-seq show the majority to be upregulated and differentially expressed (*p*.adjust ≤ 0.05, basemean >20, and a fold-change ≥1.5).

The CNS has long been described as being “immune privileged,” referencing the distinct barriers, such as the blood-brain barrier or blood-CSF barrier, that separate it from the peripheral immune system. However, following TBI, these barriers become compromised and open to peripheral immune cell invasion. Our data indicates that with age, significant upregulation of antigen-presenting genes occurs within the SON. Over-representation analysis of the 707 upregulated genes provide 30 significant biological processes (*p*.adjust < 0.01) (Supplementary File 3). Of these, the top 11 over-represented processes relate directly to antigen processing and presentation (Figure 3B). Specifically, MHCI associated antigen-processing and presentation was enriched, while MHCII genes remained unchanged. A cnetplot of selected biological processes highlights the associated genes involved (Figure 5A). Several RT1 (rat MHCs) family of genes (*Rt1-A*/*C*/*M*/*O*/*S* and *T*), which encode for the heavy α-subunits of the complex, were found to be upregulated within these pathways. Furthermore, genes involved in the antigen processing and loading of antigens to the MHC were found to be upregulated, including *Tap1/2* and *Tapbp* (Figures 5B, C). Together, in addition of several other genes, T-cell mediated cytotoxicity was found to be upregulated. Signaling pathway analysis corroborates these findings, in which antigen presentation (KEGG: rno04612) was found to be significantly enriched and activated (Figure 3C). Furthermore, the NOD-like receptor family CARD domain containing 5 (NLRC5), which acts as an MHC class I transactivator (CITA) was found to be differentially upregulated (Meissner et al., 2010). NLRC5 is a critical transcriptional regulator of many MHC class I genes within the antigen presentation pathway (Meissner et al., 2010, 2012; Downs et al., 2016). This would suggest that the upregulation of *Nlrc*5 is responsible for the activation of the MHCI pathway, as the MHCII and related CIITA were not activated.

### Characterization of methylation

**FIGURE 6:**
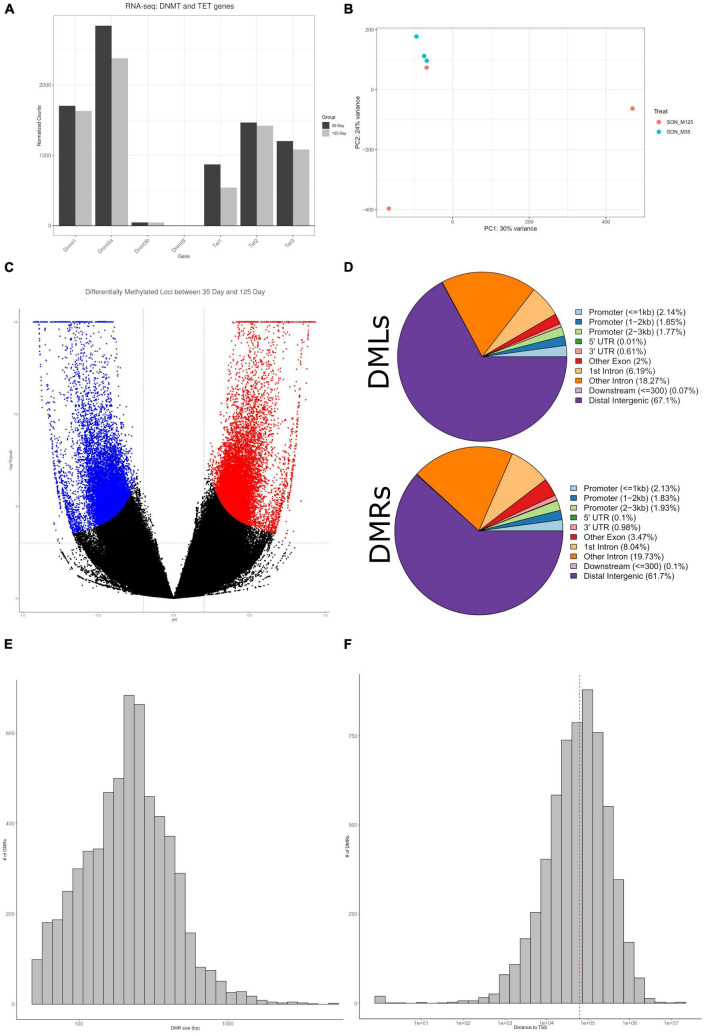
Characterization of DNA methylation. **(A)** A barplot of DNMT and TET genes from RNA-seq, plotted by normalized counts. DNMT1, DNMT3a, and Tet1/2/3, are observed to be constitutively expressed. **(B)** A PCA plot of DNA methylation indicate clustering of the 35-day old group, while the 125-day old group shows no clustering. **(C)** A volcano plot of all CpGs that were interrogated – approximately 22 million. Blue indicates statistically significant hypomethylated DMLs, red indicates statistically significant hypermethylated DMLs. **(D)** Pie charts showing the abundance of DMLs and DMRs to specific genomic features. Both DMLs and DMRs show similar profiles, the majority mapping to distal intergenic regions. **(E)** Histogram of DMR sizes. The majority of DMRs are under 1,000 bp in length. **(F)** A histogram of the distance of DMRs to TSS. Median distance to TSS is approximately 62 Kb.

We sought to investigate the role of DNA methylation to determine a mechanism that may underlie the observed maturation-dependent transcriptional changes. Our RNA-seq data indicated no differential expression of DNA methyltransferases (DNMTs), however, DNMT1 and DNMT3a were observed to be constitutively expressed (Figure 6A). Similarly, ten-eleven translocation methylcytosine dioxygenases (TETs), specifically TET1/2/3, were found to be constitutively expressed, with TET1 being differentially downregulated with age (Figure 6A). In a similar study comparing 3-month old and 18-month old rats, expression levels of DNMT1 and TET1 were found to be decreased with age, with DNTM3a and Tet2/3 showing no change (Greenwood et al., 2018). Global enzymatic activity of DNMT and TET proteins were investigated; however, we observed no detectable levels of activity (data not shown). Next, we performed long-read DNA sequencing using Oxford Nanopore sequencing to determine 5-mC DNA modifications. Approximately 22 million CpG sites were interrogated for differential methylation. Principle component analysis indicates close clustering of 35-day old replicates, whereas the 125-day old replicates exhibit no clustering (Figure 6B).

Using the DSS package, specifically the function “callDML” with a *p*-threshold < 0.001, a total of 34,435 DMLs were found – of these, 18,607 DMLs are hypermethylated and 15,828 are hypomethylated (Figure 6C and Supplementary File 4). Approximately 67% of these DMLs were found to be in distal intergenic regions, with the remaining being found within intragenic regions (Figure 6D). Annotation of these DMLs provides a total of 5,781 unique genes. For downstream analysis we took a more conservative approach to determine and analyze DMRs instead. As DMLs indicate only single CpG modifications, analyzing regions of differential methylation that contain multiple CpGs would provide greater insight into the correlation between DNA methylation and gene expression. Therefore, we performed analysis to determine DMRs, consisting of a minimum of three CpGs. From our DMR analysis, we observed 6,021 regions that were differentially methylated, with approximately 62% being found in distal intergenic regions (Figure 6D and Supplementary File 4). This provides 2,306 intragenic DMRs, that are found across 1,725 genes. Annotation of distal intergenic DMLs and DMRs is based on the nearest gene body. Due to this, DMRs can be hundreds of KBs away from genes and may not have any impact on their expression and provides poor correlation. As such, for most of our downstream analysis, we have ignored distal intergenic DMRs due to this uncertainty. The large majority of DMRs were under 1,000 bp (Figure 6E) and the median distance to transcriptional start site (TSS) was approximately 62 Kb (Figure 6F).

### Alterations to DNA methylation are enriched at axonogenesis-related genes

**FIGURE 7:**
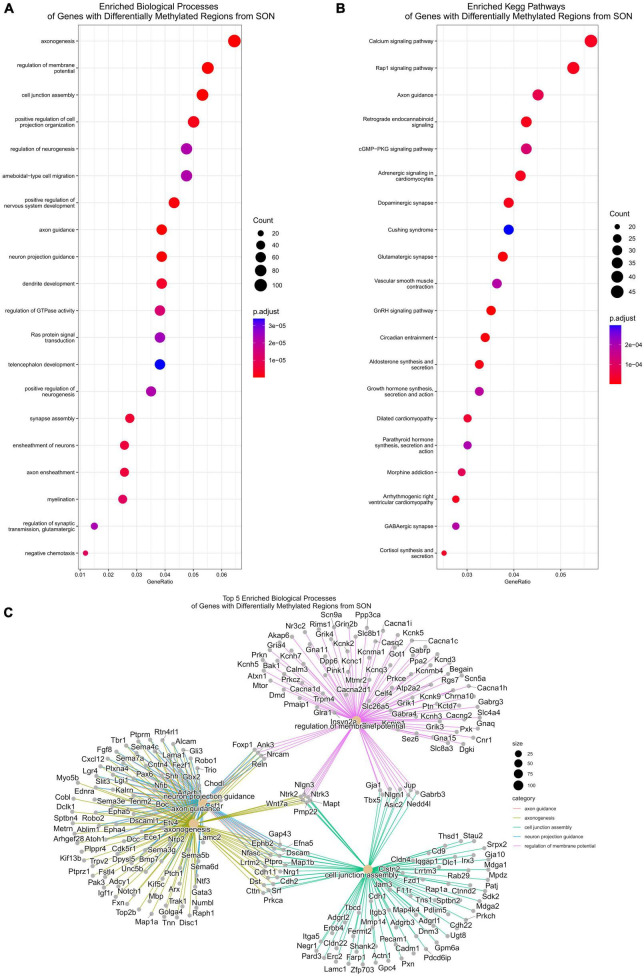
Functional analysis for differentially methylated regions. **(A)** Functional analysis of enriched biological processes of DMRs. Several biological processes related to axonogenesis are enriched, including axon guidance and regulation of neurogenesis. **(B)** KEGG pathway enrichment similarly shows enrichment of axon guidance. **(C)** A CNET plot of the top five enriched biological processes of DMRs. This shows the genes associated with axonogenesis, axon guidance, and neuron projection guidance.

We next performed functional analysis on DMRs found within intragenic regions, ignoring distal intergenic DMRs as mentioned previously. Enrichment analysis of biological processes found a total of 493 enriched processes (*p*.adjust < 0.05) (Supplementary File 5). Similar to our transcriptome data, we observed several enriched pathways related to axon plasticity (Figure 7A). By *p*.adjusted value, the top three enriched biological processes were axonogenesis (GO:0007409), axon guidance (GO:0007411), and neuron projection guidance (GO:0097485). Additional enriched biological processes of interest include regulation of neurogenesis (GO:0050767), dendrite development (GO:0016358), and positive regulation of cell projection organization (GO:0031346) (Figure 7A). Likewise, analysis of KEGG pathways identified a total of 76 enriched pathways (*p*.adjust < 0.05), with axon guidance being significantly enriched (rno04360) (Figure 7B and Supplementary File 5). As such, similar family of genes that were found to be differentially expressed were observed to be differentially methylated (Figure 7C). These include members of the semaphorin family and their respective plexin receptor family, and the ephrin family and their receptors (Figure 7C). In contrast, while we found a robust upregulation of MHCI pathway DEGs, functional analysis of DMRs found no significant changes in related biological processes.

**FIGURE 8:**
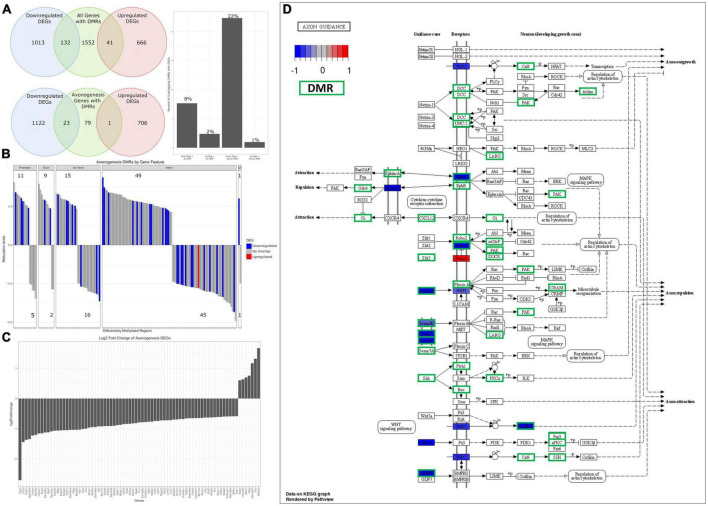
Analysis of overlapping DMRs and DEGs related to axonogenesis. **(A)** A Venn diagram of overlapping DEGs and DMRs. Of the genes with DMRs, 8% overlap with downregulated DEGs (barplot), with 2% overlapping with upregulated DEGs. Of axonogenesis genes with DMRs, 22% overlap with downregulated DEGs (barplot) and 1% overlap with upregulated DEGs. **(B)** A barplot plotting all axonogenesis DMRs and their methylation status – hypermethylated is defined as greater than 0, hypomethylated less than 0. Promoters and exons trend toward hypermethylated, while intronic methylation is split. DMRs that overlap with DEGs have their expression status overlaid – blue indicates DMRs that overlap with downregulated DEGs, red indicates DMRs that overlap with upregulated DEGs. **(C)** A barplot of differentially expressed axonogenesis genes from RNA-seq, plotted by log2FC. The majority can be seen to be downregulated. **(D)** Differential expression and differentially methylation data is overlaid on the axon guidance KEGG pathway. This indicates that the downregulation of semaphorins and ephrins correspond to differential methylation.

As we saw statistically significant changes in methylation of axonogenesis genes and the axon guidance pathway, we aimed to determine the extent in which differential methylation correlates to changes in gene expression. To do this, we assessed the number of genes with DMRs that overlapped with DEGs – the total number of genes with DMRs was 1,725, with 132 overlapping with downregulated DEGs (∼8%) and 41 overlapping with upregulated DEGs (∼2%) (Figure 8A). Similarly, we assessed the number of axonogenesis genes with DMRs that overlapped with DEGs – there were a total of 103 axonogenesis genes with DMRs, with 23 overlapping with downregulated DEGs (∼22%) and 1 overlapping with upregulated DEGs (∼1%) (Figure 8A). This indicates that compared to baseline (total DMRs), the overlapping of axonogenesis DMRs with DEGs are enriched, and specifically that axonogenesis DMRs correlated stronger with downregulated DEGs compared to upregulated DEGs. These data suggest that differential methylation of axonogenesis genes is negatively impacting gene expression.

To obtain insight into the directionality of methylation and its impact on gene expression of axonogenesis genes, we plotted the axonogenesis DMRs and their methylation status and overlaid the respective differential gene expression data. The 103 axonogenesis genes that contain DMRs correspond to a total of 154 DMRs – promoter and exon methylation trend toward hypermethylation, while intron and 3′ UTR are more equally hyper and hypo methylated (Figure 8B). This was expected as promoter methylation correlates to downregulation of gene expression, whereas other intragenic regions are much more variable. Our data highlights that the vast majority of axonogenesis DEGs were downregulated, and that DEGs that contain DMRs contain promoter and exon hypermethylation, with heterogenous methylation of other intragenic regions (Figures 8B, C). Likewise, overlaying DEG and DMR data onto the Axon Guidance (rno04360) KEGG pathway highlights specific signaling pathways (Figure 8D). Here we observed that the semaphorin and ephrin signaling pathways shared several overlapping regions of differential expression and methylation, as well as the Slit/Robo pathways. Furthermore, this highlights the extensive differential methylation that occurred throughout the axon guidance pathway, and although there are some that do not overlap with statistically significant DEGs, they may still play a role in the overall inhibition of the pathway.

### Motif analysis reveals AP-1 transcription factors correlate to hypomethylated distal intergenic regions

Our next step was to determine how methylation with age may affect the binding of specific transcription factors by analyzing motif enrichment. In our previous analysis, we ignored distal intergenic DNA methylation as their association to genes could not be well-established. As our aim is to determine motif enrichment and thus the potential of enrichment of regulatory elements such as enhancer sites in intergenic regions, we have incorporated all DMRs into the analysis. Furthermore, previously we were not able to separate genes with DMRs into hyper- and hypo-methylated as many genes were heterogeneously methylated. In contrast, for our motif analysis, we have determined motif enrichment profiles for hyper- and hypo-methylated DMRs – this is important as transcription factor binding can be promoted or inhibited based on the methylation state (Yin et al., 2017; Héberlé and Bardet, 2019).

**FIGURE 9:**
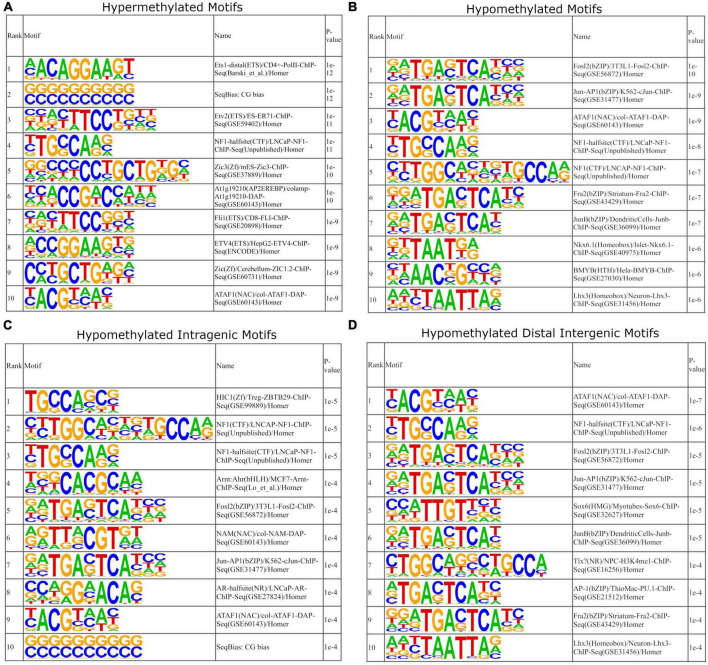
Motif enrichment analysis of DMRs. DMRs were separated by hyper and hypomethylation. **(A)** The top 10 motifs of hypermethylated DMRs. Hypermethylated motifs correspond to transcription factors that have diverse and broad roles in cellular development. **(B)** The top 10 motifs of hypomethylated DRMs. Several DMRs correspond proteins that comprise the AP-1 transcription factor complex – Jun and Fos specifically. **(C,D)** Separation of hypomethylated motifs into intragenic and distal intergenic motifs, respectively. This indicates that motifs for AP-1 transcription factor complex are more enriched within hypomethylated distal intergenic motifs.

Whole genome motif analysis provided differing results between hyper- and hypo-methylated DMRs. Within the top 10 enriched motifs of hyper-methylated DMRs, many transcription factors have wide variety of functions and do not confer to specific biological roles (Figure 9A). For example, the E26 transformation-specific (ETS) transcription factors (Ets1, etv2, fli1, and ETV4) are part of a large family that are present throughout the body and regulate diverse biological processes such as cellular senescence, cell differentiation, and proliferation (Sharrocks, 2001). Similarly, zinc finger of the cerebellum (ZIC) family mediate diverse processes that are critical during early development (Hatayama and Aruga, 2018).

In contrast, motif analysis of hypomethylated DMRs suggest motif enrichment of immune related transcription factors that may explain the upregulation of genes observed in the antigen presentation pathways. Within the top 10 enriched motifs, several are components of the AP-1 transcription factor complex (Figure 9B). AP-1 is a heterodimer composed of the c-Fos, c-Jun, ATF, and JDP protein families, of which FOS (Fosl2 and Fra2) and Jun (Jun-AP1 and JunB) were enriched (Eferl and Wagner, 2003). Members of the AP-1 complex, specifically FOS and Jun have been shown to be sensitive to DNA methylation, such that DNA methylation suppresses their ability to bind and that they preferentially bind to unmethylated DNA (Mann et al., 2013; Spruijt et al., 2013; Yin et al., 2017; Héberlé and Bardet, 2019). Next, we separated hypomethylated intragenic and distal intergenic DMRs and performed the same enrichment analysis to determine if these motifs are potentially enriched within the gene body or at regulator elements. For hypomethylated intragenic DMRs, we saw a reduction in AP-1 related motifs, with Fosl2 and Jun-AP1 motifs remaining (Figure 9C). Within the hypomethylated distal intergenic DMRs, we saw an increase in AP-1 related motifs – Fosl2, Jun-AP1, JunB, AP-1, and Fra2 (Figure 9D). This data indicates that AP-1 motifs are being hypomethylated at distal intergenic sites with age. Previous studies have shown that AP-1 often exerts gene expression regulation by binding to enhancer sites (Bejjani et al., 2019). As such, this data suggests that the hypomethylation of AP-1 related motifs would promote the expression of many downstream genes that are involved in cellular stress responses including the observed upregulation of MHCI and T-cell activation genes through the binding of enhancer sites (Foletta et al., 1998; Arias et al., 2010; Kulpa et al., 2013; Atsaves et al., 2019).

## Discussion

It is a well-known phenomenon that neuronal plasticity declines in the mammalian CNS with age; however, the mechanisms that underlie this process are poorly understood. This loss in the capacity for neurons to regenerate or reorganize axons is emphasized following TBI. This was shown in our previous studies – following unilateral axotomization of MCNs of the SON, the contralateral, non-lesioned MCNs undergo axonal sprouting in 35-day old rats. Whereas this capacity for neuronal plasticity is lost in 125-day old rats. Thus, our goal was to determine the transcriptomic and DNA methylomic alterations that occur between these two age groups in uninjured rats. Through this, we have determined key changes in the gene expression of axonogenesis and immune-related pathways, and how methylation may directly and indirectly regulate these profiles.

### The loss of growth factors with age and their role in neuronal plasticity

Our data has shown that with age, several important growth factors that participate in neuron survivability and plasticity were downregulated. Brain-derived neurotrophic factor (BDNF) belongs to a family of neurotrophins that play crucial roles in these functions (Huang and Reichardt, 2001). The primary receptor in which BDNF exerts its neurotrophic affect is via TrkB (*Ntrk2*), and like many of the growth factors mentioned (FGF and IGF), it signals through the PI3K-AKT pathway, which was observed to be inhibited (Figure 3C and Supplementary Figure 2). In our previous research utilizing organotypic cultures, we have shown that CNTF promotes axonal sprouting of MCNs in the SON via the PI3K-Akt pathway, suggesting that growth factors such as BDNF may play a similar role (Askvig and Watt, 2015). BDNF has been shown to exert neuroprotective and regenerative sprouting effects on neuronal populations following injury in a variety of model systems in both the CNS and PNS (Deng et al., 1999; Mamounas et al., 2000; Almeida et al., 2005; Liao et al., 2015; Kowiański et al., 2018; McGregor and English, 2019; Gustafsson et al., 2021). In fact, studies involving spinal cord hemisection in rats have shown contralateral axon sprouting and not ipsilateral regeneration, and is promoted by the exogeneous application of BDNF (Vavrek et al., 2006; Cao et al., 2021). Similarly, in our previous experiments, the contralateral, non-lesioned MCNs in young rats (35 days) underwent axonal sprouting, whereas this phenomenon is lost in the older rats (125 days) (Watt and Paden, 1991; Watt et al., 1999; Askvig and Watt, 2019). These data suggest that the downregulation of BDNF that we observed in the 125-day old rats may play a significant role in preventing axonal sprouting.

Fibroblast growth factors are well-known for their diverse functionality throughout the body (Turner and Grose, 2010). More relevantly, FGFs have important roles in the nervous system that range from neuron repair to axon guidance (Guillemot and Zimmer, 2011). Our data indicated the downregulation of several FGF’s – 10/12/13/18, and interestingly, the upregulation of FGF2. FGF10 has been shown to be important for the initiation of neurogenesis, reduce neuroinflammation following TBI, aid in axon guidance, and activate the neuronal PI3K-Akt signaling pathway and inhibit the TLR4/NF-κB dependent neuroinflammation following spinal cord injury (Sahara and O’Leary, 2009; Chen et al., 2017; Hou et al., 2020; Liu et al., 2020). FGF12/13/18 are less studied in the nervous system but aid in developmental processes; however, more recent work has indicated that FGF13 may play an important role in axon regeneration through axon formation and growth cone initiation via microtubule stabilization (Wu et al., 2012; Li J. et al., 2018; Klimaschewski and Claus, 2021). The upregulation of FGF2 is interesting as it has been shown to be strongly involved in neuroprotection and repair (Yoshimura et al., 2001). Furthermore, it has been shown that FGF2 promotes collateral axon branching (Szebenyi et al., 2001). In our model system, the mechanism for the increased axon termini stemming from the contralateral SON following lesion has not yet been investigated – it is unknown whether the increase in these termini are due to increased terminals from existing axon branches or new collateral axon branches are formed that elongate and terminate in the posterior pituitary. This future direction could shed light on the role of FGF2 within our model system. Similar to FGFs, IGFs and their associated binding proteins (IGFBPs) have shown to regulate neuronal survivability and axon regeneration (Nieto-Estévez et al., 2016; Allard and Duan, 2018). IGF1 has been highly studied and has shown to be an important factor for axon growth and cell survival primarily through the PI3K-Akt pathway (O’Kusky et al., 2000; Laurino et al., 2005; Dupraz et al., 2013; Liu et al., 2017).

We observed the downregulation of several growth factors and signaling genes within the PI3K-Akt pathway of the older rats. The key functionality of many of these growth factors fall within the roles of neuron survival, growth cone initiation, axon branching, and axon guidance. As such, our data suggests that a key mechanism for the loss of axon sprouting in our model system is due to the loss of these important growth factors.

### The loss of axonogenesis with age and the role of DNA methylation

Our data indicated a downregulation of several biological processes pertinent to the loss of neuronal plasticity and axon regeneration (Figures 3A, C). Of note, these included the biological processes axonogenesis (GO:0007409), axon guidance (GO:0007411), and neuron projection guidance (GO:0097485), and the KEGG signaling pathway axon guidance (rno04360). Within the CNET plot of axonogenesis, its observed that two major families are affected, the semaphorins and ephrins (Figures 4A-C). Of the differentially expressed semaphorins, *Sema3a*/*c*/*d*/*e*/*f*, *Sema4d*, S*ema5a*/*b*, and *Sema6a* were downregulated, while *Sema3b* was upregulated. There are two primary receptors for SEMAs – plexins, which were constitutively expressed, but not differentially, and neuropilins, which were downregulated (*Nrp1*/*2*). The semaphorin family, as a whole, is classified as having diverse roles but are known to act as guidance cues for axon repulsion and attraction (Koncina et al., 2013; Hu and Zhu, 2018). One of the most well-studied are class 3 semaphorins, which have been shown to act as axon guidance molecules, and are upregulated following injury (De Winter et al., 2002; Hashimoto et al., 2004; Hou et al., 2008; Sharma et al., 2012; Bribián et al., 2014). *Sema4d* was first perceived as an immune semaphorin due to its lymphocytic role, but similar to the other semaphorins, it shares many roles in axon outgrowth and guidance (Wang et al., 2001; Kumanogoh and Kikutani, 2003; Masuda et al., 2004; Tasaka et al., 2012; Peng et al., 2017).

Like semaphorins, ephrins function as axon guidance factors and aid in neural development and plasticity following CNS injury (Klein, 2004; Yang et al., 2018). Ephrins are grouped into two classes, ephrin-A and ephrin-B. Our data indicates that of the ligands, only members of ephrin-B are downregulated (*Efnb2* and *Efnb3*), whereas, of the receptors, only members of ephrin-A are downregulated (*Epha3*/*5*/*6*/*7*). In general, the binding of ephrin ligands to receptors are exclusive to their respective class; however, there seems to be limited cross-binding between the two classes (Kullander and Klein, 2002; Himanen et al., 2004; Pitulescu and Adams, 2010). Ephrins play a greater role in axon repulsion than attraction in regards to axon guidance, and are observed to be upregulated following CNS injury (Miranda et al., 1999; Moreno-Flores and Wandosell, 1999; Rodger et al., 2001; Willson et al., 2002; Bundesen et al., 2003; Nikolakopoulou et al., 2016; Yang et al., 2018). In fact, this axon repulsion by ephrins may be an important factor that leads to axon inhibition at glial scars (Chen et al., 2013). However, as noted before, our model system indicates axonal sprouting to occur from the contralateral side of the lesion, and thus not inhibited by any glial scarring. Therefore, the downregulation of ephrins may be a loss of repulsive guidance, akin to guidance during development, and not inhibitory as seen at glial scars.

As we have shown, there is a robust downregulation of several families of axonogenesis genes which follows the well-established phenomenon of a loss of neural plasticity with age and consequently poor recovery rates following TBI (Burke and Barnes, 2006; Dhandapani et al., 2012). One of our goals for this study was to determine a mechanism that may explain age-related changes to gene expressions. Our genome-wide DNA methylation investigation determined that many biological processes involved in neural plasticity are differentially methylated. While we focused on axonogenesis (GO:0007409), several other relevant biological processes that were differentially methylated include: axon guidance (GO:0007411), neuron projection guidance (GO:0097485), regulation of neurogenesis (GO:0050767), dendrite development (GO:0016358), and positive regulation of cell projection organization (GO:0031346) (Figure 7A and Supplementary File 5). There are many mechanisms for regulating gene expression, so we wanted to determine the extent in which DNA methylation was enriched within our DEGs. Surprisingly, approximately 10% of all genes that contain DMRs overlapped with DEGs – 8% overlapped with downregulated, and 2% with upregulated DEGs. However, when we subset DMR genes to include only axonogenesis genes, we see that this percentage of overlapping greatly increases – 22% of axonogenesis DMR genes overlap with downregulated DEGs (Figure 8A). Methylation of the promoter region is well-known to be correlated with downregulation of gene expression. On the other hand, introns are less correlative and thus, the methylation status and its impact on expression more variable; nonetheless, studies have shown that differential methylation is associated with changes in gene expression and may promote alternative splicing (Illingworth and Bird, 2009; Anastasiadi et al., 2018; Li S. et al., 2018; Tabish et al., 2019). Within the axonogenesis biological process, the majority of genes were downregulated (Figure 8C), however, the methylation status of DEGs were quite variable (Figure 8B). Promoter methylation of axonogenesis genes correlated to downregulated genes, whereas intronic methylation showed a nearly equal split between hypermethylation and hypomethylation of downregulated genes (Figure 8B). To gain a signaling pathway view of differential methylation and expression, we overlaid this data to the axon guidance KEGG pathway (Figure 8D). The pathway shows a large number of DMR genes, with semaphorins and ephrins being differentially methylated. These data suggest that differential methylation correlates to the downregulation of axonogenesis-related genes and therefore an inhibition of axon regeneration.

### The role of MHCI in neural plasticity and regeneration

With age, it is common to see increases in inflammatory markers within the CNS, from increases in chemokines and cytokines to gliosis (Ferrucci and Fabbri, 2018; Stephenson et al., 2018; Mészáros et al., 2020). However, we saw a complete lack of inflammatory profiles within our sequencing data. Interestingly, there was a distinct MHCI signature within our RNA-seq data, while the MHCII pathway was unchanged (Figures 3B, 5). MHCI is expressed by all nucleated cells and interact with and activate CD8^+^ T cells via T-cell receptors (TCRs) (Neefjes et al., 2011). In the CNS, MHCI and the receptor PirB have been implicated in preventing functional recovery following injury (Adelson et al., 2012; Bochner et al., 2014). Reduced astrogliosis has been shown to occur in CNS injury in β2m knockout mice, a component of the MHCI complex (Cartarozzi et al., 2019). Furthermore, depleting cytotoxic CD8^+^ T cells promotes neurological recovery following TBI (Daglas et al., 2019). These data suggest that the increase in MHCI would promote inflammatory and cytotoxic environments following TBI through increased gliosis and peripheral invasion of CD8^+^ T cells. While this would occur locally at the site of injury, it is possible that this inflammatory and immune response could affect the contralateral side if not properly contained, thus preventing axonal sprouting in the older rats. Nevertheless, ongoing research has shown that MHCI has an important non-immunological role in neural plasticity and development (Cebrián et al., 2014).

MHCI has been shown to drastically increase and peak during early development and gradually decrease into early adulthood as it is an important factor for the establishment and refinement of synaptic networks through pruning (Huh et al., 2000; Bilousova et al., 2012; Liu et al., 2013; McAllister, 2014; Tetruashvily et al., 2016; Shen et al., 2019). Later in age, MHCI appears to again gradually increase throughout adulthood and into advanced age as it is critical for the maintenance of neuronal structural complexity in the brain (Starkey et al., 2012; Lazarczyk et al., 2016). Thus, MHCI is an important factor the establishment, refinement, and then maintenance in neural networks. Together, this suggests that in early age, with increased growth factors, the opposing force of MHCI allows for *refined* growth of axons (via pruning) important for accurate innervation; however, in later stages of life, this balance appears to favor increased MHCI in order to maintain existing network connections but at the cost of regenerative ability following injury. Our data follows this existing trend and suggests that the upregulation of MHCI would inhibit axonal sprouting even in the uninjured, contralateral MCNs of older rats.

DNA methylation interrogation was performed to determine a cause for this upregulation in MHCI. As noted before, a critical regulator of MHCI genes, *Nlrc5*, was also upregulated (Meissner et al., 2012; Jongsma et al., 2019). However, we sought to investigate a mechanism for this increased expression. Our initial DNA methylation analysis showed no indication of differential methylation within this biological process or those related to it. We next performed motif enrichment analysis. Within the hypermethylated motifs, there were minimal correlative outcomes to axonogenesis or MHCI and immune-related processes. However, within the hypomethylated motifs, we observed several enriched motifs for AP-1 transcription factors within the top 10 enriched motifs (Figure 9B). This enrichment of AP-1 transcription factor motifs was further increased in hypomethylated distal intergenic DMRs (Figure 9D). AP-1 is a transcription factor complex that is a heterodimer of proteins that belong to the c-Fos, c-Jun, ATF, and JDP families. It regulates gene expression following a variety of stimuli and is associated with regulating immunity, specifically related to T-cell activation (Atsaves et al., 2019). AP-1 complex proteins were found to be constitutively expressed within our system, so the opening and closing of binding sites via DNA methylation provides a mechanism for regulating their function. However, the exact mechanism for AP-1 binding and regulating gene expression is still not clear. While it can act as a local switch by binding near to the transcriptional start site of genes, recent studies suggest that AP-1 often operates by binding to distal enhancer sites (Kwasnieski et al., 2014; Zanconato et al., 2015; Nguyen et al., 2016; Bejjani et al., 2019). Furthermore, several studies have shown that AP-1 binding is inhibited by hypermethylation at or near to their binding motif (Mann et al., 2013; Yin et al., 2017; Héberlé and Bardet, 2019). Our data indicates that there is enrichment of AP-1 binding motifs at hypomethylated distal intergenic sites, and prior studies indicate these sites to be enhancers. These data suggest that AP-1 promotes the upregulation of MHCI genes through distal enhancer binding which in turn is regulated by DNA methylation.

### Similarities to other models of SON plasticity

The SON has been known to be a highly plastic region that undergoes structural, morphological, and molecular changes in response to a number of stimuli. There are several studies that have explored the differential gene expression of age and dehydration. Our data shared many similarities with these studies. In Elsamad et al. (2023), the authors identified similar differential transcriptome profiles between adult (3-month-old) and aged (18-month-old) rats in differing hydrated states, in which PI3K-Akt, axon guidance, and antigen presentation pathways were found to be differentially expressed between ages of euhydrated animals (Elsamad et al., 2023). This suggest that these changes persist throughout age. Similarly, in another study that performed dehydration for 72 h in adult (11 weeks) rats, the PI3K-Akt pathway was found to be enriched (Pauža et al., 2021). Specific gene similarities also persist across these age groups, including UCN, which was also found to have an age-dependent association with hypertension in MCNs of the SON and PVN (Martin et al., 2021; Elsamad et al., 2023). Additionally, several semaphorins were also found to be downregulated in later age groups, while oxytocin was found to be upregulated (Elsamad et al., 2023).

## Conclusion

In summary, we sought to investigate the transcriptome and methylome of the SON within an age-dependent model system of axonal sprouting. Our data indicates that the loss of axonal sprouting with age is due to two primary factors: the loss of axonogenesis related genes including key growth factors, and the enhanced expression of MHCI genes. Furthermore, we have shown that differential methylation occurs at an enriched rate at axonogenesis genes and may act to repress their expression. Additionally, MHCI gene expression appears to be regulated through distal intergenic methylation at enhancer sites for AP-1. Our study is the first to describe the methylome of the SON in an age-dependent manner, and to our knowledge, it is the first to associate distal enhancer site methylation of AP-1 binding and MHCI gene expression. In future studies we aim to investigate these factors within the context of injury and determine the role of histone modifications within our system. The goal of this and future projects are to further the body of research aimed toward CNS recovery following TBI. By providing insight into molecular mechanisms that underlie age-dependent axonal sprouting, we gain a better understanding of the processes that determine a favorable or detrimental prognosis in patients with TBI.

## Data availability statement

The data presented in the study are deposited in the Gene Expression Omnibus (GEO) online repository, accession number GSE231617 and can be found at: https://www.ncbi.nlm.nih.gov/geo/query/acc.cgi?acc=GSE231617.

## Ethics statement

The animal study was reviewed and approved by the University of North Dakota (UND) Institutional Animal Care and Use Committee – protocol number 1905-7.

## Author contributions

DT contributed to the conception, methodology, data analysis, investigation, and the writing of the original draft. AO contributed to the investigation. CB contributed to the review and revision of the manuscript. JW contributed to the conceptualization, review and revision of the manuscript, and supervision. All authors contributed to the article and approved the submitted version.
