# Supplementary figures and images for: Transcriptome and methylome of the supraoptic nucleus provides insights into the age-dependent loss of neuronal plasticity
Source: Front Aging Neurosci. 2023 Aug 30;15:1223273. doi: 10.3389/fnagi.2023.1223273 (PMC10498476; doi:10.3389/fnagi.2023.1223273)

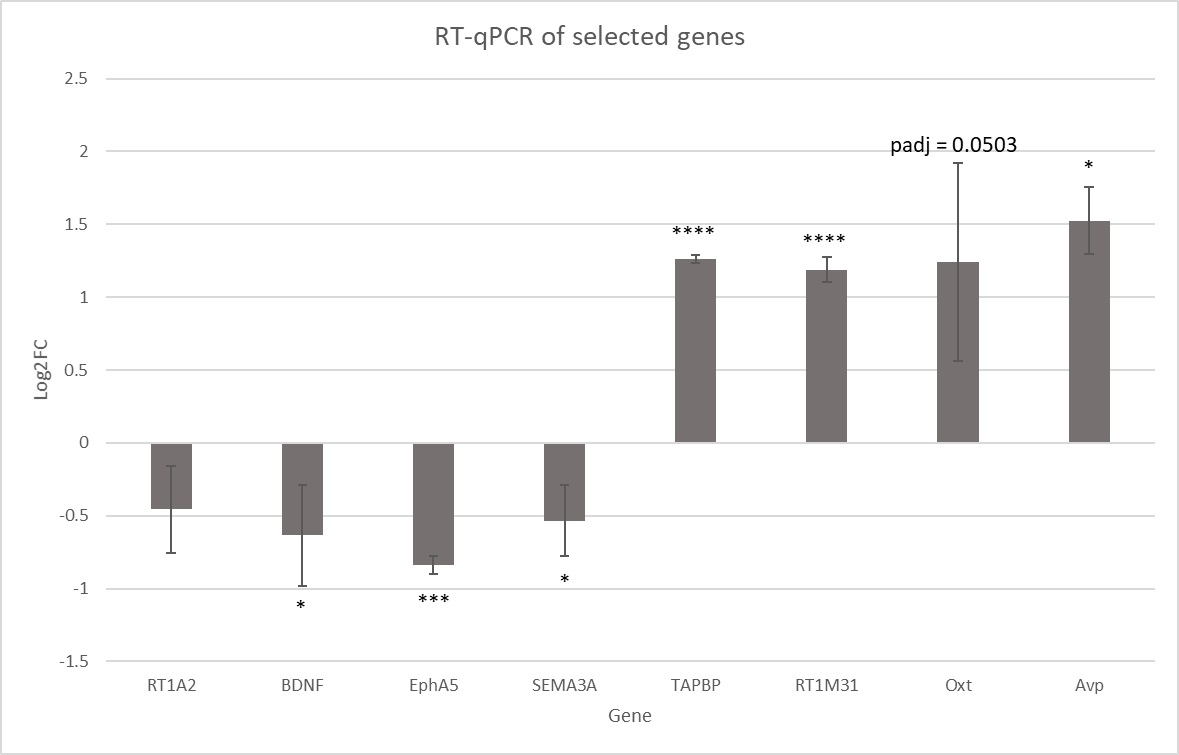

Supplement: Supplementary file 2 [file Image_1.JPEG]

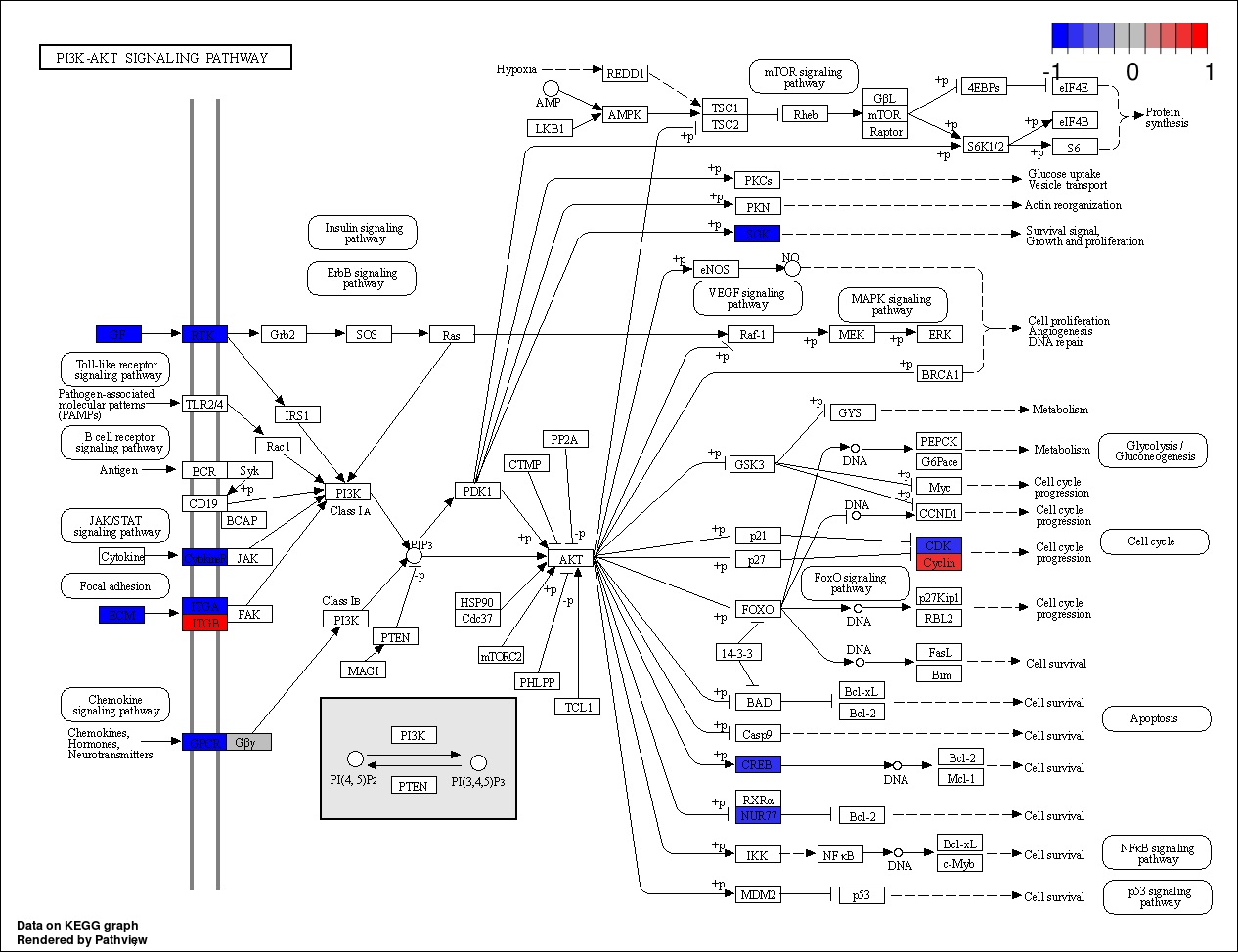

Supplement: Supplementary file 3 [file Image_2.JPEG]
